# Determinants of out-of-district health facility bypassing in East Java, Indonesia

**DOI:** 10.1093/inthealth/ihaa104

**Published:** 2021-01-27

**Authors:** Nuzulul Kusuma Putri, Ratna Dwi Wulandari, Robeth Jabbar Syahansyah, Karen A Grépin

**Affiliations:** The Airlangga Centre for Health Policy, Kampus C Mulyorejo, Surabaya 60115, Indonesia; Department of Health Policy and Administration, Faculty of Public Health Universitas Airlangga, Kampus C Mulyorejo, Surabaya 60115, Indonesia; The Airlangga Centre for Health Policy, Kampus C Mulyorejo, Surabaya 60115, Indonesia; Department of Health Policy and Administration, Faculty of Public Health Universitas Airlangga, Kampus C Mulyorejo, Surabaya 60115, Indonesia; Dr. Soetomo General Academic Hospital, Jl. Mayjen Prof. Dr. Moestopo No.6-8, Gubeng, Surabaya 60286, Indonesia; Division of Health Economics, Policy, and Management, School of Public Health, University of Hong Kong, 7 Sassoon Road, Sandy Bay, Hong Kong

**Keywords:** access, bypassing, district, health insurance

## Abstract

**Background:**

Several large-scale reforms, including policies aimed at achieving universal health coverage, have been implemented to overcome health disparities in Indonesia. However, access to health services remains unequal. Many people ‘bypass’ health services in their home district to access health services in neighbouring districts, even though their health insurance does not cover such services. This study aims to identify the factors that are associated with this out-of-district bypassing behaviour.

**Methods:**

We surveyed 500 respondents living in the outermost districts of East Java province. We used data on education, income, district, age, gender, household size, district accessibility, insurance coverage status and satisfaction with health facilities in the home district and logistic regression analysis to model the predictors of out-of-district health facility bypassing.

**Results:**

The most important predictors of the bypassing behaviour were education and poor access to health facilities in the home district. Open-ended data also found that the most important reason for seeking care in another district was mostly geographic. In contrast, health insurance coverage does not appear to be a significant predictor.

**Conclusions:**

Education and geographic factors are the main predictors of out-of-district bypassing behaviour, which appears to be how border communities express their health facility preferences. Local and central governments should continue their work to reduce inequality in access to health facilities in Indonesia's geographically challenged districts.

## Introduction

Indonesia is one of the most populous countries in the world; however, relative to other large countries, its population is widely dispersed across >17 000 islands. This geographic feature poses an enormous challenge to the central government of Indonesia in its attempts to achieve universal health coverage (UHC); in particular, efforts to ensure equal access to high-quality health services. In 2016, for example, Indonesia had only 1.12 hospital beds per 1000 people, far from the World Health Organization's (WHO) recommendation of 5 beds per 1000 people.^1^ In response, the central government has designated 143 of 514 districts (27.8%) as being disadvantaged, frontier or outermost districts (DFO districts), which they have targeted to receive additional resources to reduce inequalities in access to health services.

Over the past few decades, the Indonesian Ministry of Health (MOH) has implemented several large-scale reforms aimed at reducing disparities in health service access and improving health equity. First, launched in 1968, the central government established the *Puskesmas* as the backbone of the public health system, which ensures that at least one primary health centre is available in each subdistrict. Many *Puskesmas*, however, especially those in the DFO districts, have found it challenging to attract and retain health workers, especially doctors, which has led to a persistent maldistribution of health workers across the country.^2,3^ Hence the central government decentralized the responsibility for healthcare delivery down to the districts starting in 1999,^4^ assuming a more decentralized healthcare system would lead to a more flexible allocation of resources and more accessible health services.^5–7^ However, even though decentralization led to increased utilization of outpatient health services in Indonesia, the overall levels of inequalities in the use of health facilities increased between the more affluent and less affluent parts of the country.^8,9^ Indonesia also expanded coverage of health insurance, in particular for the poor and near-poor, through health financing reforms. Since 2014, *Jamkesmas* (community health insurance) and *Jamkesda* (district health insurance), the old health financing schemes that varied between districts, were replaced by a new mandatory national social health insurance scheme, *Jaminan Kesehatan Nasional* (*JKN*). Despite these reforms, access to health services in Indonesia remains unequal between urban and rural areas.^10^ Remote regions also suffer from an inadequate supply of quality health services.^11^

In Indonesia, given the decentralization policy and how health insurance is financed, citizens are supposed to use the health services made available to them in their home district. Specifically, those whose contributions are subsidized by the district government are supposed to use the health facilities owned by their district government. However, in practice, it has been observed that many people ‘bypass’ health services in their home district in favour of health services in neighbouring or nearby districts. In such cases, households are expected to pay for health services out of pocket, as these services are not eligible for coverage under their district health insurance plan. Therefore the decision to seek care outside of the home district is likely an expression of preference for quality health services or other factors. The existence of this behaviour, and the lack of studies on this topic, motivated the MOH to undertake this study to better understand the factors associated with what we call out-of-district health facility bypassing behaviour.

In other international contexts, studies have explored the related phenomenon of health facility bypassing, wherein people decide to use a health facility located further away from them than a more proximal health facility. It is believed that health facility bypassing represents a strong expression by citizens of a preference for high-quality health services.^12^ Indeed, studies have shown that facility quality,^13,14^ as well as financial barriers^15^ and the perceived competency of health workers,^13–15^ all contribute to higher rates of facility bypassing. Previous studies, however, have not specifically investigated the phenomenon of out-of-district health-seeking behaviour, nor in the context of decentralization, gaps this article aims to address. In this article we analyse the factors that were associated with out-of-district bypassing behaviour among people living in border districts of East Java, Indonesia, using household survey data.

The setting for our study, East Java province, is the second most populated province in Indonesia. It has 38 districts and 4 of them are considered DFO districts. Although each subdistrict has at least one *Puskesmas*, many subdistricts face many geographic challenges (e.g., mountains). The East Java provincial government also established village health posts (*Ponkesdes*) to extend the reach of the *Puskesmas* service into more remote villages. Despite this, most of *Ponkesdes* report insufficient health personnel.^16^ Moreover, studies have documented important inequalities in access to health services within the province.^17^

## Methods

### Sample selection

**Figure 1.**
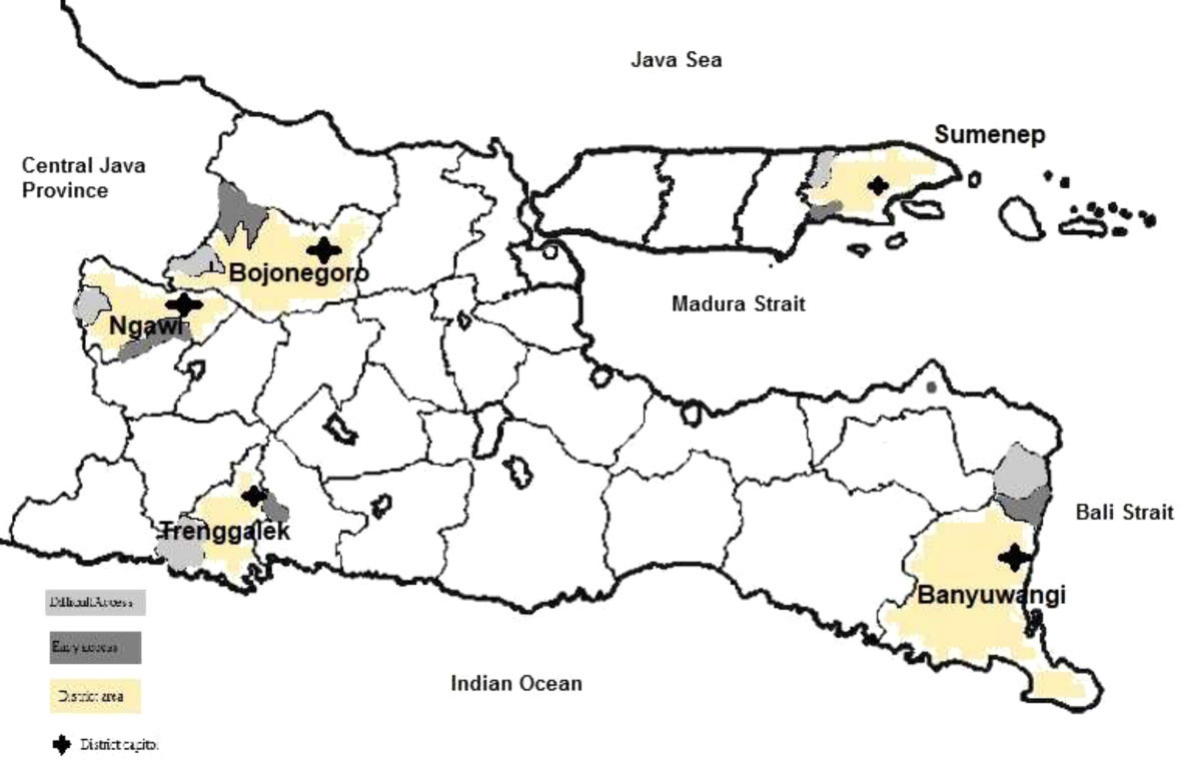
The sample districts.

To investigate the phenomenon of out-of-district health facility bypassing, we surveyed 500 respondents living in five districts in East Java. The sampled districts were all located in the outermost regions of the province and were selected among 38 districts in coordination with the East Java provincial government (see Figure 1). We purposely selected five districts that varied in terms of their border characteristics. Ngawi and Bojonegoro districts are adjacent to each other and both also border Central Java province, a more affluent province. It was hypothesized that people living in those districts would have more choice in health facilities due to their relative proximity to another province. Sumenep district is located on a separate island while still being part of the East Java province and does not share a land border with any other district. Trenggalek district was selected, as it is adjacent to another district by land and also is bordered by the sea. Banyuwangi district was chosen due to its proximity to Bali. Even though a strait separates it, Banyuwangi is closer to Bali rather than to the East Java provincial capital.

To control for physical access within the district, in each district, two subdistricts were selected: one subdistrict with easy access and one subdistrict with difficult access to the district capital. To choose the subdistricts, we asked the District Health Office in each district to recommend a subdistrict in their district that has easy access and one subdistrict with difficult access to the district capital as defined by public transportation networks. Subdistricts with difficult access were defined as those that do not have a main road connecting it to the district capital and/or any direct public transportation to reach the district capital. In each selected subdistrict we then selected 50 respondents using a random walk strategy. To randomly select households within each subdistrict, enumerators started at the subdistrict health office. They selected houses at regular intervals of five houses to the left and right side of the subdistrict office. We selected this strategy to ensure that households located both near and far from the subdistrict health office would be included in the sample. As all of the subdistrict offices are located in the town centre, households closer to the subdistrict office would have better access to primary health facilities. As there were 10 selected subdistricts (5 subdistricts with and without difficult access in 5 districts), our final potential sample size was 500 respondents. The survey was conducted from May to July 2015, 1.5 y after the *JKN* program was launched in Indonesia. We developed the survey measurements and consulted an Indonesian linguist to ensure that the questions were easy to understand. We hired enumerators with a public health educational background and trained them to understand all of the variables to be collected in the survey.

### Data

We asked respondents whether they had ever travelled to other districts for healthcare (for themselves or another household member) within the last 3 months, which we use as the measure of out-of-district health facility bypassing. We also collected data on education, income, district, age, gender, household size, district access level, insurance coverage status and satisfaction toward health facilities in their home district, which we use as additional variables in our model. District access level refers to whether the household lived in one of the subdistricts with difficult public transportation access based on the recommendation of the District Health Office. Satisfaction was defined as the respondent's impression of health facilities in their home district. It has been shown in other contexts that patient satisfaction has been associated with the decision to seek care.^13,14,18^ We made a satisfaction scale based on the service dimension used by the national government in assessing the public satisfaction index of public organizations in Indonesia. A total of 16 satisfaction questions, each with a 5-point rating scale (mean 0.76 [standard deviation 0.07]), were asked to respondents. We tested the scale reliability of our instrument by estimating the Cronbach's α, which yielded a sufficiently high coefficient (Cronbach's α=0.86). To calculate the satisfaction score, we summed the level of satisfaction across the 16 questions and then divided the sum by 80 (16×5) to get an overall satisfaction score. We controlled for the income of households, as this is an important predictor of health service utilization in other contexts.^19–21^ To measure income, we asked the respondents how much money was earned by their family over the past month and asked them to place themselves into five potential categories of income.

For a small number of observations we had missing data on a few variables, such as the age of respondents, household size, satisfaction score and education. We imputed the missing data by replacing the missing observations with the mean value of the sample (i.e., for age we used 41.91 y, for the household size we used four people, for satisfaction score we used 0.76, for education we used elementary school level). We present results with imputed data but also provide our estimates without imputed data in Appendix Table 1, the results of which are very similar.

For all respondents who reported they or a household member used a health facility outside of their home district in the past month, we also asked respondents an open-ended question to list the factors that made them bypass the health facilities in their home district. They could answer freely and provide any potential reason for their decision, including more than a single reason for this decision. To summarize these data, we used inductive coding by grouping responses with the same themes by first reading all of the responses, then creating categories of response types and then applying the codes to the full sample.

### Data analysis

We used multiple binary logistic regression analysis to model the predictors of out-of-district health facility bypassing using variables that are important predictors of health facility bypassing and health service utilization in other contexts. The main outcome in this study was bypassing behaviour, which we defined as a binary variable based on whether the respondent had travelled to another district for healthcare (for themselves or another household member) within the last 3 months. Our base model included data on levels of education, monthly income and a district-level fixed effect. Studies have shown that females and older patients are more likely to bypass health facilities,^13,22^ so we also tested the impact of gender and age in one of our specifications. We also included household size, since *JKN* coverage is a function of household size; specifically, *JKN* coverage is defined at the household level, and bigger households are expected to pay a higher contribution to the scheme. We then tested the policy-relevant variables to determine whether they predict seeking care outside of the district. First, we tested the impact of whether the respondent lived in one of the more difficult-access subdistricts within the sampled district. Second, we tested the impact of having health insurance on the propensity to seek care outside of the district. Third, we tested whether satisfaction with past healthcare interactions in their district played a role in the decision to seek care outside of their home district. Finally, we tested the impact of all of the policy-relevant variables jointly on the propensity to seek care outside of the district.

## Results

In total, 443 respondents completed the survey (88.6% response rate) and the demographics of our sample are presented in Table 1. Of the completed questionnaires, 51.2% were from respondents in easy-access subdistricts and 48.8% were from respondents living in the difficult-access subdistricts. Slightly more than the majority of the respondents were female (60.0%), only 32% had health insurance, 66% had low monthly income that was >1 000 000 Indonesian rupiah (IDR) and 62.1% of the sample had only an elementary school education or less. The overall level of satisfaction index toward healthcare service in the home district was high; the mean of satisfaction score was 0.76, suggesting relatively high levels of satisfaction with health facilities in their home district.

**Table 1.** Sample summary statistics

Variables	N	n	% or mean	Standard deviation
Travelled outside home district to access health services	443	293	66.1%	0.47
Lived in district with difficult geographical access to health facility	443	216	48.8%	0.50
Had health insurance	443	143	32.3%	0.47
Knew whether this health provider is in their district:				
Government-owned health facility				
Village midwife	443	377	85.1%	0.36
*Polindes*	443	110	24.8%	0.43
*Ponkesdes*	443	7	1.6%	0.12
*Puskesmas pembantu*	443	193	43.6%	0.50
*Puskesmas*	443	353	79.7%	0.40
District hospital	443	337	76.1%	0.43
Private health facility				
Nurse	443	310	70.0%	0.46
General practitioner	443	323	72.9%	0.44
Private clinic	443	132	29.8%	0.46
Maternity clinic	443	129	29.1%	0.45
Private hospital	443	216	48.8%	0.50
Home district (% of total)	443			
Banyuwangi		73	16.5%	
Bojonegoro		100	22.6%	
Ngawi		70	15.8%	
Sumenep		100	22.6%	
Trenggalek		100	22.6%	
Monthly family income (IDR, % of total)	443			
<1 000 000		292	65.9%	
1 000 000–<2 000 000		106	23.9%	
2 000 000–<3 000 000		28	6.3%	
3 000 000–<4 000 000		7	1.6%	
≥4 000 000		10	2.3%	
Education (% of total)	421			
Did not attend formal education		77	18.3%	
Did not pass elementary school		10	2.4%	
Elementary school		166	39.4%	
Junior high school		74	17.6%	
Senior high school		68	16.2%	
University		26	6.2%	
Age of respondent (years)	442		41.91	12.58
Sex (% of total)	443			
Male		175	39.5%	
Female		268	60.5%	0.49
Household size (persons)	430		3.84	1.35
Satisfaction score toward health facility in home district	437		0.76	0.07

Overall rates of out-of-district health facility bypassing were quite high in the sample: 66% of the respondents reported that they or a member of the household had travelled outside of their home district to access health services over the last 3 months. Among those who bypassed, most bypassed to access primary healthcare service in another district (85.3%), 13.3% bypassed to access *Puskesmas* managed by another district, while only 4.4% bypassed to access a private hospital in another district. The highest out-of-district health facility bypassing rate was reported in Ngawi district (94.3% decided to bypass), while the lowest was in Banyuwangi district (20.5%). Based on a simple cross-tabulation, even though the bypassing behaviour is high among the population living in difficult-access (62.5%) and easy-access areas, we found a higher proportion of the people living in easy-access areas bypassed (69.6%).

In Table 2 we present results from our regression models that investigate the determinants of out-of-district health facility bypassing using logistic probability estimation. Our base model included only socio-economic variables (education level, monthly income, home district). Overall, higher education levels and residing in Ngawi district were associated with significantly higher odds of bypassing.

**Table 2.** Predictors of out-of-district health facility bypassing

Variables	Base model	Model 1	Model 2	Model 3	Model 4	Model 5
Did not pass elementary school	1.439* (0.802)	1.442* (0.828)	1.770** (0.836)	1.346* (0.812)	1.451* (0.801)	1.672* (0.865)
Elementary school	1.716*** (0.362)	1.670*** (0.370)	2.155*** (0.407)	1.695*** (0.364)	1.695*** (0.369)	2.074*** (0.422)
Junior high school	1.592*** (0.437)	1.551*** (0.464)	2.145*** (0.495)	1.553*** (0.440)	1.592*** (0.440)	2.123*** (0.540)
Senior high school	1.647*** (0.460)	1.609*** (0.489)	2.231*** (0.520)	1.614*** (0.462)	1.639*** (0.462)	2.221*** (0.573)
University	0.592 (0.610)	0.540 (0.646)	1.272* (0.666)	0.573 (0.614)	0.787 (0.638)	1.518** (0.766)
IDR 1 000 000–<2 000 000	0.312 (0.330)	0.361 (0.333)	0.363 (0.337)	0.308 (0.329)	0.326 (0.329)	0.409 (0.338)
IDR 2 000 000–<3 000 000	0.208 (0.541)	0.150 (0.548)	0.437 (0.567)	0.247 (0.546)	0.400 (0.570)	0.635 (0.615)
IDR ≥4 000 000	0.773 (1.176)	0.740 (1.177)	0.921 (1.183)	0.862 (1.184)	0.697 (1.180)	0.927 (1.200)
Banyuwangi	−1.669*** (0.374)	−1.589*** (0.391)	−1.737*** (0.384)	−1.734*** (0.383)	−1.610*** (0.384)	−1.709*** (0.420)
Bojonegoro	1.317*** (0.371)	1.335*** (0.375)	1.435*** (0.380)	1.331*** (0.372)	1.321*** (0.372)	1.483*** (0.384)
Ngawi	2.390*** (0.595)	2.268*** (0.596)	2.985*** (0.662)	2.382*** (0.593)	2.368*** (0.609)	2.854*** (0.676)
Sumenep	0.995*** (0.360)	0.936** (0.369)	1.225*** (0.373)	1.009*** (0.362)	1.064*** (0.372)	1.290*** (0.400)
Age		−0.003 (0.011)				0.001 (0.012)
Female		−0.272 (0.260)				−0.260 (0.265)
Household size		−0.095 (0.091)				−0.083 (0.094)
District access level			0.817*** (0.309)			0.863*** (0.333)
Health insurance				−0.225 (0.275)		−0.321 (0.296)
Satisfaction toward health facility in home district					0.561 (1.939)	0.672 (2.021)
Constant	−1.195*** (0.399)	−0.497 (0.829)	−2.144*** (0.546)	−1.098*** (0.416)	−1.666 (1.540)	−2.170 (1.796)
Observations	415	401	415	415	409	395

Values presented as mean (standard error).

*p<0.1, **p<0.05, ***p<0.01.

‘Did not attend formal education’ was omitted in the education section. ‘IDR <1 000 000’ was omitted in the monthly income section. The income level at IDR 3 000 000–<4 000 000 predicts success perfectly and was dropped and six observations were not used. Trenggalek was omitted in the home district section.

We then tested a specification that also included age, sex and household size in addition to the base model variables. The results show that these three variables were not correlated with out-of-district bypassing behaviour. We then tested the impact of the policy-relevant variables on the propensity of respondents to seek care outside of their district. When we included a binary variable to capture whether the respondent lived in a district with difficult access to health services, we found that more educated people and those who lived in a district with difficult access were more likely to bypass health facilities in their own district. When we tested the impact of health insurance, we found no significant effect of having health insurance on the propensity to seek healthcare outside of the home district. Satisfaction with health facilities in the district also did not affect the propensity to seek care outside of the district. When we included all of the variables in our final specification, we found that having a formal education and living in a difficult-access district significantly increased bypassing behaviour. Education was also a strong predictor of bypassing behaviour: respondents with a senior high school education had 39.1% (odds ratio [OR] 1.647 [95% confidence interval {CI} 0.222 to 0.560]) higher odds to bypass their home district. Respondents residing in Ngawi district were found to have the highest odds to bypass: residence in this district was associated with 48.0% (OR 2.390 [95% CI 0.271 to 0.689]) higher odds to travel outside their district to access healthcare. Living in a district with difficult access also led to 14.5% (OR 0.863 [95% CI 0.0383 to 0.252]) higher odds to bypass.

To better understand why people decided to bypass the healthcare facility in their home district we utilized the open-ended survey data. We summarize these data in Table 3. The most common reason people gave as to why they sought care outside of their home district was because the nearest health facility to their home was located in another district (39.9%). The second most common response given was due to the perceived higher levels of satisfaction with healthcare providers in health facilities outside of their district. People also reported bypassing to access a provider that they felt ‘fit’ with them better than in their home district (22.9%). Economic factors were the third most important reason; specifically, 17.7% of bypassers reported that out-of-district facilities were more affordable, despite the lack of insurance coverage, than health facilities in their own district, perhaps due to lower transportation costs.

**Table 3.** Stated reasons for bypassing health facility in the home district

Reason	n	%
The out-of-district health facility was nearer to their home	117	39.9
Felt more ‘fit’ with the out-of-district health provider	67	22.9
The out-of-district health facility provided more affordable healthcare	52	17.7
The out-of-district health providers were more competent	13	4.4
The out-of-district health facility had faster response	13	4.4
The out-of-district health facility was open after hours	10	3.4
The out-of-district health facility can be reached by public transportation	7	2.4
The health provider in their home district was often absent	7	2.4
The health technology out of district was more complete	5	1.7
Administrative procedures in the home district are complicated	2	0.7
Total	293	100.0

## Discussion

To our knowledge, our study is the first study to investigate the phenomenon of out-of-district health facility bypassing behaviour. Our findings suggest that despite efforts to improve access to health services through decentralization and through expanding coverage of health insurance, the decision to seek care outside of the home district is a common occurrence in border districts in Indonesia.

We find that geographic access appears to be the main reason why people living in border areas decide to go out of their district to seek health services. Perhaps unsurprisingly, people who bypass health facilities in their district may be better serviced by health facilities in other districts. This is consistent with previous studies of general health facility bypassing that have shown that travel distance from the patient's home to the healthcare facility location is an important predictor of which facilities are utilized.^14^

We also found that out-of-district health facility bypassing behaviour is more likely to occur among more educated people. Being more educated may provide people with more means and the ability to express their preference for the health facility they seek care from.

Previous studies have also suggested that quality of care is an important predictor of the decision to bypass closer health facilities.^15,23^ Unlike previous studies, we found that the satisfaction towards health facilities in the home district had no significant effect on the prevalence of bypassing behaviour.^14,24^ While this may be related to challenges associated with the measurement of satisfaction, it also supports our main finding that geographical access is the most important factor that predicts out-of-district health facility bypassing behaviour.

We also found that health insurance coverage does not predict out-of-district health facility bypassing, which is consistent with findings from a study that found people tend to use nearer health facilities when the travel costs exceed the monetary value of their benefits covered under their health insurance.^25^ Other studies, however, have reported that people with health insurance are more likely to bypass a more proximal health facility.^13,24,26^

While our study has shed some light on an important research question that has not seen a lot of attention in the literature, our study has several limitations. We were unable to measure the severity of the health conditions of people who bypassed health facilities vs those who did not, which also likely affects this behaviour. Our study also purposely selected districts and subdistricts based on geographic factors and therefore our results are only representative of similar populations and not of the general Indonesian or even East Java population. This study also highlights the challenges of trying to capture factors that may explain variations in bypassing across contexts. For example, variations in the policies implemented by and the financial positions of local governments are other potential factors that could explain this behaviour, but we did not measure this in our study. Moreover, the strategy of local governments building infrastructure in remote areas also differs, but again, we did not have data to control for this variation.

## Conclusion

We found the overall prevalence of out-of-district bypassing to be very high in our study, despite the institutional factors that suggest that using health services should be more difficult for people living in other districts. The primary factors we found to be associated with out-of-district bypassing appear to be poor access to health facilities in the home district. The behaviour also appears to be more prevalent among more educated people, suggesting that bypassing can be challenging to many and that these socio-economic factors may allow people to express their preferences for health facilities more freely. Interestingly, health insurance coverage does not appear to be an important predictor of whether people seek care within their district. Since geography is the fundamental reason for bypassing, the central governments should work to find ways to continue to narrow the gap in access that exists between districts, especially those with more challenging geography. Districts could map their existing health infrastructure and overlay this with information of out-of-district bypassing to better understand where this behaviour is most likely to occur. The central government may also wish to explore how to allocate resources to best increase access in these areas, for example, by allowing people to use their health insurance in neighbouring districts where such decisions may make it easier for them to access health services.

## Data Availability

All data generated or analysed during the current study are available at DOI 10.17605/OSF.IO/KZGQX.

## Appendix 2

**Table 1A.** Regression result (standard error) after imputation

Variables	Base model	Model 1	Model 2	Model 3	Model 4	Model 5
Did not pass elementary school	1.463* (0.805)	1.554* (0.835)	1.803** (0.840)	1.372* (0.816)	1.463* (0.805)	1.737** (0.873)
Elementary school	1.743*** (0.362)	1.752*** (0.365)	2.185*** (0.404)	1.722*** (0.364)	1.745*** (0.364)	2.206*** (0.410)
Junior high school	1.594*** (0.440)	1.566*** (0.463)	2.175*** (0.496)	1.555*** (0.444)	1.595*** (0.440)	2.233*** (0.536)
Senior high school	1.645*** (0.463)	1.612*** (0.487)	2.260*** (0.522)	1.610*** (0.465)	1.645*** (0.464)	2.341*** (0.569)
University	0.565 (0.612)	0.506 (0.645)	1.290* (0.667)	0.547 (0.615)	0.564 (0.612)	1.430* (0.736)
IDR 1 000 000–<2 000 000	0.360 (0.328)	0.379 (0.329)	0.396 (0.336)	0.356 (0.328)	0.360 (0.328)	0.411 (0.338)
IDR ≥2 000 000–<3 000 000	0.246 (0.540)	0.214 (0.550)	0.466 (0.566)	0.285 (0.545)	0.245 (0.542)	0.507 (0.591)
IDR ≥4 000 000	0.858 (1.161)	0.828 (1.160)	0.967 (1.176)	0.939 (1.169)	0.857 (1.162)	1.103 (1.192)
Banyuwangi	−1.719*** (0.373)	−1.803*** (0.385)	−1.786*** (0.383)	−1.783*** (0.382)	−1.722*** (0.383)	−1.947*** (0.414)
Bojonegoro	1.317*** (0.373)	1.344*** (0.377)	1.443*** (0.381)	1.330*** (0.373)	1.316*** (0.374)	1.513*** (0.386)
Ngawi	2.592***	2.511***	3.103***	2.585***	2.596***	3.066***
	(0.583)	(0.584)	(0.644)	(0.581)	(0.590)	(0.656)
Sumenep	1.014***	0.964***	1.247***	1.027***	1.011***	1.304***
	(0.361)	(0.369)	(0.373)	(0.363)	(0.366)	(0.392)
Age		−0.005 (0.011)				0.002 (0.012)
Female		−0.264 (0.258)				−0.218 (0.261)
Household size		−0.080 (0.092)				−0.063 (0.093)
District access level			0.852*** (0.305)			0.981*** (0.327)
Health insurance				−0.221 (0.275)		−0.402 (0.293)
Satisfaction toward health facility in home district					−0.074 (1.918)	0.382 (1.999)
Constant	−1.222*** (0.400)	−0.505 (0.827)	−2.201*** (0.541)	−1.127*** (0.417)	−1.165 (1.525)	−2.194 (1.782)
Observations	436	436	436	436	436	436

*p<0.1, **p<0.05, ***p<0.01.

‘Did not attend formal education’ was omitted in the education section. ‘IDR <1 000 000’ was omitted in the monthly income section. The income level at IDR 3 000 000–<4 000 000 predicts success perfectly. This was dropped and seven observation were not used. Trenggalek was omitted in the home district section. We imputed the missing data by replacing the missing observations with the mean value of the sample.

**Table 2A.** Marginal effects

Variables	No imputation data, mean (95% CI)	With imputation data, mean (95% CI)
Did not pass elementary school	0.307* (0.0277 to 0.586)	0.296* (0.0326 to 0.559)
		
Elementary school	0.370*** (0.242 to 0.498)	0.365*** (0.250 to 0.480)
		
Junior high school	0.377*** (0.215 to 0.539)	0.368*** (0.219 to 0.518)
		
Senior high school	0.391*** (0.222 to 0.560)	0.383*** (0.227 to 0.539)
		
University	0.281* (0.0227 to 0.538)	0.247* (0.0114 to 0.482)
IDR 1 000 000–<2 000 000	0.0681 (−0.0389 to 0.175)	0.0632 (−0.0356 to 0.162)
IDR 2 000 000–<3 000 000	0.103 (−0.0775 to 0.283)	0.0771 (−0.0902 to 0.244)
IDR ≥4 000 000	0.143 (−0.170 to 0.457)	0.155 (−0.122 to 0.431)
Banyuwangi	-0.288*** (−0.414 to −0.161)	−0.303*** (−0.415 to −0.190)
Bojonegoro	0.250*** (0.131 to 0.368)	0.235*** (0.125 to 0.346)
Ngawi	0.480*** (0.271 to 0.689)	0.477*** (0.290 to 0.663)
Sumenep	0.217*** (0.0906 to 0.344)	0.203*** (0.0881 to 0.317)
Age	0.000246 (−0.00380 to 0.00429)	0.000322 (−0.00334 to 0.00399)
Female	−0.0437 (−0.131 to 0.0432)	−0.0338 (−0.113 to 0.0455)
Household size	−0.0140 (−0.0448 to 0.0167)	−0.00985 (−0.0382 to 0.0185)
District access level	0.145** (0.0383 to 0.252)	0.152** (0.0558 to 0.249)
Health insurance	-0.0541 (−0.151 to 0.0430)	−0.0625 (−0.151 to 0.0261)
Satisfaction toward health facility in home district	0.113 (−0.553 to 0.779)	0.0594 (−0.550 to 0.669)
N	395	436

*p<0.05, **p<0.01, ***p<0.001.
